# Population genetics of *Cryptosporidium parvum* subtypes in cattle in Poland: the geographical change of strain prevalence and circulation over time

**DOI:** 10.1186/s12917-022-03328-y

**Published:** 2022-07-06

**Authors:** Agnieszka Kaupke, Artur Rzeżutka

**Affiliations:** Department of Food and Environmental Virology, National Veterinary Research Institute, Al. Partyzantów 57, 24-100 Puławy, Poland

**Keywords:** *Cryptosporidium parvum*, Cattle, Prevalence, Molecular detection, Subtyping, Population genetics

## Abstract

**Background:**

*Cryptosporidium parvum* (*C. parvum*) is a cosmopolitan parasite that infects various livestock animals including cattle. Microsatellite typing tools for identification of *C. parvum* subtypes are currently employed to better understand the species-specific epidemiology of cattle cryptosporidiosis. The aim of this study was to analyse the population genetics of *C. parvum* strains infecting cattle and recognise geographical distribution and time-span correlations in subtype prevalence in Poland. In total, 1601 faecal samples were collected from 2014 to 2018 from healthy cattle from dairy, meat and mixed breeds at the age of 1 week to 4 months. The 267 farms visited were randomly selected and represented all Polish provinces. PCR–RFLP based identification of *C. parvum* at the 18 small subunit ribosomal RNA (SSU rRNA) locus was performed, followed by strain subtyping by GP60-PCR.

**Results:**

The overall prevalence of *C. parvum* in Polish cattle was estimated at 6.2% (100/1601). Animals below the age of 1 month were the major host for this parasite. Excluding one breed, that of dairy-meat mixed, there were no significant differences observed between breed and presence of *C. parvum* infections (95% TPI_All breeds_: 1.67–73.53%; POPR = 0.05—0.95). Infected animals were detected in 15 out of 16 Polish provinces, with significant regional prevalence diffrences (Kruskal–Wallis rank sum test, Kruskal–Wallis χ^2^ = 13.46, *p* < 0.001). When the population genetics of *C. parvum* strains were analysed, 11 parasite subtypes from the IIa and IId genetic families were identified. Compared to other parasite strains, IIaA17G1R1 and IIaA17G2R1 appeared at statistically significantly higher frequency (F-test, *F* = 3.39; *p* = 0.0003). The prevalence of *C. parvum* subtypes in cattle was breed-related (Chi-squared test, χ^2^ = 143.6; *p* < 0.001).

**Conclusions:**

The analysis of the population genetics of *C. parvum* subtypes showed that strains from the IIa subtype family predominated in the tested cattle population. However, relations in changes of subtype prevalence and circulation over time were observed. They were associated with the disappearance of some strains and emergence of new variants from the same genetic family in different geographical locations.

## Highlights

*C. parvum* subtypes from the IIa allele family predominated in the tested cattle. The prevalence of *C. parvum* subtypes in cattle was breed-related. Dynamicity in the population *C. parvum* strains circulating in cattle was shown.

## Background

Among the parasitic diseases of cattle, cryptosporidiosis is of major importance [1, 2]. Symptomatic cryptosporidiosis is mainly associated with *C. parvum* infections [3–6]. The prevalence of *C. parvum* infections in cattle herds in Europe has been found to range from 1.2 to 100% and has more often been observed in diarrhoeic animals [7, 8]. Outside Europe, they have also occurred with varied prevalences ranging from 5 to 50% in North America [9, 10], 3.6 to 97.3% in South America, 0.3 to 93% in Africa [11, 12] and 3.4 to 93% in Asia [13, 14].

In Poland, the first attempt to assess the prevalence of *Cryptosporidium* infection in cattle was undertaken by Rzeżutka et al. in 2013 [15]. The overall prevalence of *Cryptosporidium* infection in the tested animal population was estimated at 17%; however, *C. parvum* was not the most frequently detected parasite species, being discovered in 5.1% of cases. The analysis of the microsatellite GP60 region of the *C. parvum* genome has revealed a various number of circulating subtypes belonging to three subtype families, which were IIa, IId, and IIl before 2014 [16]. Other investigations are in accordance regarding the circulation of the IIa subtype family, it having been the most frequently detected *C. parvum* subtype in European cattle [17–19]. Reports in the literature also note that the IId and IIl genetic families have been found less frequently [3, 4, 20, 21], and the pattern in Poland was similar as found by Kaupke and Rzeżutka [16], *C. parvum* strains of IIa subtype predominating compared to the sporadically appearing IId and IIl strains. In research up to the time of writing, the *C. parvum* IIaA17G1R1, IIaA17G2R1 and IIaA15G2R1 strains have been most often identified. Of note is that IIaA15G2R1 has also appeared as the dominant subtype in several European cattle populations [19, 20, 22–27]. *C. parvum* can cause infections in various hosts including humans [28]. A major role in parasite transmission is played by cattle, which are the natural reservoir of the parasite [29–32]. It has been shown that *C. parvum* infections in humans are more common in areas of intensive livestock farming [4].

Although numerous studies have been conducted that aimed at identification of *C. parvum* subtypes of bovine origin, our knowledge of population genetics of strains of this parasite and their geographical prevalence is still not complete. Furthermore, the population structure and subtype distribution in livestock as they relate to host age also require analysis. The aim of this study was to analyse the population and geographical distribution of *C. parvum* strains infecting cattle in Poland. Additionally, changes of subtype prevalence and circulation over time in the population of Polish cattle were investigated.

## Results

### Detection and age-related ocurrence of *C. parvum* infections in cattle

The 18 SSU rRNA gene fragment was successfully amplified in 725 out of 1601 cattle faeces samples. After *Nde*I digestion of all *Cryptosporidium*-positive samples, a restriction pattern of 18 SSU rRNA amplicons characteristic of *C. parvum* was shown for 100 DNA samples. Two samples contained a mixture of different parasite sequences including that of *C. parvum*. Their subsequent treatment with *Mbo*II confirmed identification of *C. parvum* and revealed the presence of *C. bovis*. The overall prevalence of *C. parvum* in Polish cattle was estimated at 6.2% (100/1601). *C. parvum* infections were detected in all age groups of cattle at 11.9% (63/527) frequency at 1–4 weeks, 4.9% (23/464) at > 4–8 weeks and 2.3% (14/610) at > 8–16 weeks. A relationship was observed between animal age and frequency of infection (Chi-squared test, χ^2^ = 1439.2, *p* < 0.001). Animals below the age of 1 month were the major host for this parasite and the number of *C. parvum*-positive cattle decreased with animal age.

### Breed related prevalence of *C. parvum*

The sampled animals mainly represented dairy breeds (74.8%), meat and mixed dairy–meat breeds being far smaller proportions (respectively 14.5% and 10.7%). Animals of Polish Black and White Holstein Friesian (HO), Limousie (LM), mixed exclusively meat breed (MM), Simentaler (SM), mixed exclusively dairy breed (MS) and mixed dairy–meat breed (MDM) breeds were found positive for *C. parvum* DNA, the majority of infections (74/1601) being detected in dairy cattle of HO breed (Table 1). There were no significant differences observed between cattle breeds (95% true prevalence estimate (TPI)_All breeds_: 1.67–73.53%; POPR = 0.05–0.95) in appearance of *C. parvum* infections, with one exception: animals of MDM breed differed from the other breeds (95% TPI_MDM breeds_: 2.79–9.25%; POPR ≤ 0.05 and POPR ≥ 0.95). Mixed *C. parvum* and *C. bovis* infections were not associated with any particular breed (95% TPI_All breeds_: < 0.0001–42.54%).

**Table 1:** Prevalence of *C. parvum* infections in cattle of different breeds in Poland

*Cryptosporidium* species	Cattle breeds^a^															
	HO (*n* = 1112)	LM (*n* = 72)	MM (*n* = 145)	SM (*n* = 93)	ZB (*n* = 22)	RW (*n* = 61)	MO (*n* = 10)	NCB (*n* = 28)	BS (*n* = 1)	AN (*n* = 6)	CH (*n* = 7)	RP (*n* = 7)	MS (*n* = 22)	JE (*n* = 1)	MDM (*n* = 12)	Total
*C. parvum*	74	6	6	4	-	-	-	-	-	-	-	-	1	-	7	98
*C. parvum and C. bovis*	2	-	-	-	-	-	-	-	-	-	-	-	-	-	-	2

^a^*HO* Polish Black and White Holstein*–*Friesian*, LM* Limousie, *MM* Mixed meat bread, *SM* Simentaler, *ZB* Polish Black and White, *RW* Polish Red and White Holstein*–*Friesian*, MO* Montbeliarde, *NCB* Black and White lowland, *BS* Brown Swiss, *AN* Aberdeen Angus, *CH* Charolaise, *BB* Belgian Blue, *RP* Polish Red, *MS* Mixed without meat breed, *JE* Jersey, *MDM* mixed dairy—meat breeds

### Geographical distribution of *C. parvum* infections

**Fig. 1:**
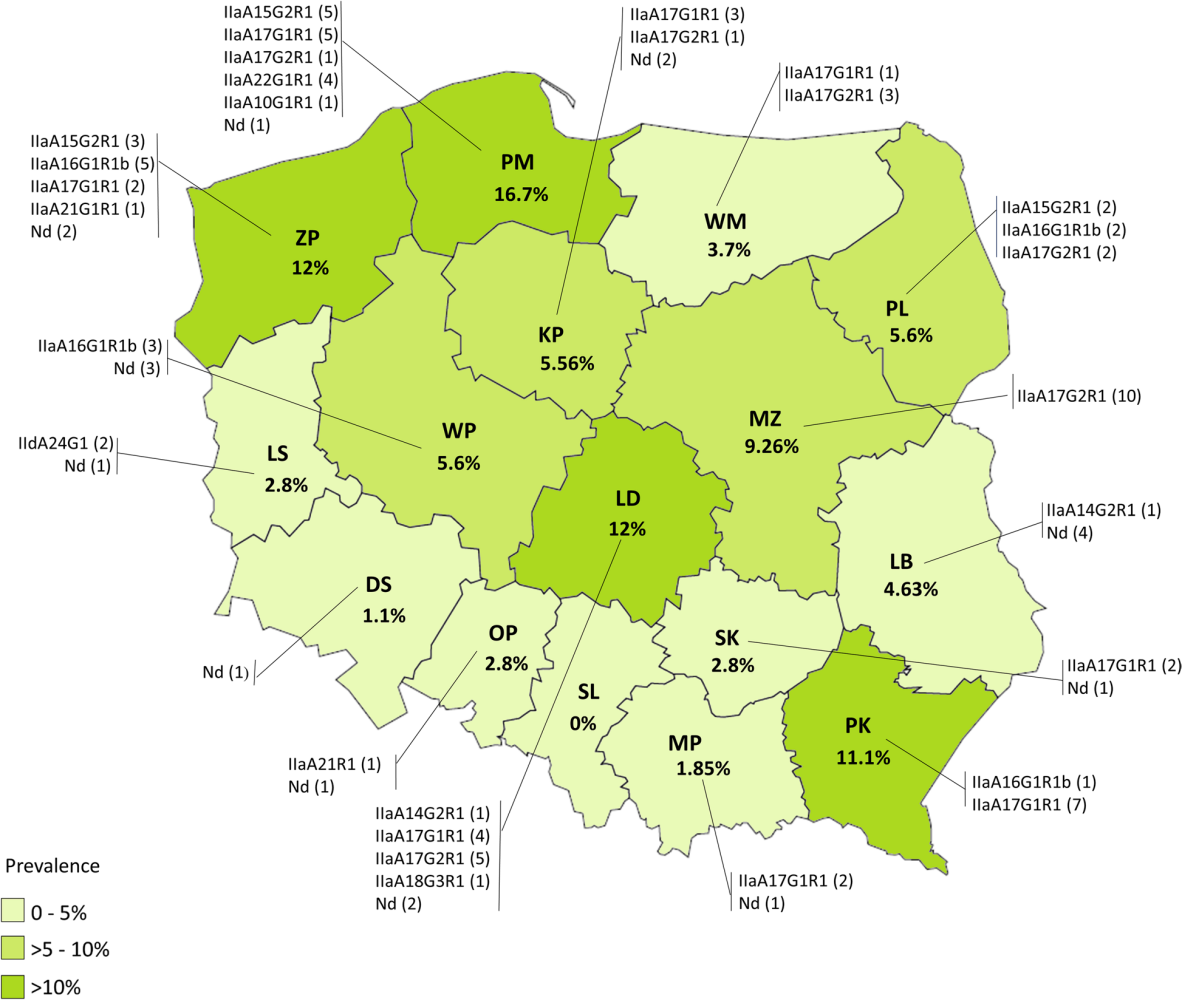
The geographical distribution of *C. parvum* strains in Poland. Values in brackets indicate at number of detected parasite strains of particular subtypes. The percentage values shown on the map indicate at *C. parvum* prevalence in particular province. *DS* Dolnośląskie, *KP* Kujawsko-Pomorskie, *LB* Lubelskie, *LD* Łódzkie, *LS* Lubuskie, *MP* Małopolskie, *MZ* Mazowieckie, *OP* Opolskie, *PK* Podkarpackie, *PL* Podlaskie, *PM* Pomorskie, *SK* Świętokrzyskie, *SL* Śląskie, *WM* Warmińsko-Mazurskie, *WP* Wielkopolskie, *ZP* Zachodniopomorskie, *Nd* Not determined

The infected animals were reared on 44 (16.5%) out of the 267 monitored farms and were located over the whole country. All evaluated farms in the Zachodniopomorskie (ZP) and Dolnośląskie (DS) provinces appeared to be *C. parvum* positive. None of the tested cattle from Śląskie province (SL) herds was *C. parvum* positive. Infections with this parasite were detected in farms located across 15 Polish provinces with varied prevalences (Table 2, Fig. 1). Significant differences in regional prevalences of *C. parvum* infections between the groups of provinces (high, medium and low) in Poland were shown (Kruskal–Wallis rank sum test, Kruskal–Wallis χ^2^ = 13.46, *p* < 0.001). Pomorskie (PM), ZP, Łódźkie (LD), Podkarpackie (PK) and Mazowieckie (MZ) provinces were characterised by higher prevalence of infections (16.7 ≥ 9.3%) and in descending order of prevalence the remaining provinces were Wielkopolskie (WP), Podlaskie (PL), Kujawsko-Pomorskie (KP), Lubelskie (LB), Warmińsko-Mazurskie (WM) (medium prevalence < 5.6–3.7%) and Lubuskie (LS), Opolskie (OP), Świętokrzyskie (SK), Małopolskie (MP), DS and SL (low prevalence < 2.8%). The highest frequency of parasite infections was in PM at 16.7% and the lowest in DS province at 1.1%, where only 1 out of 15 farms kept infected animals (Table 2).

**Table 2:** The number of *C. parvum* positive animals in particular age groups and Polish provinces

Province^a^	All	Number of tested animals			Positive animals	Prevalence (%)
		in age groups (weeks)				
		1–4	> 4–8	> 8–16		
DS	90	-	-	1	1	1.1
KP	108	3	2	1	6	5.56
LB	108	3	2	-	5	4.63
LD	108	8	4	1	13	12
LS	108	3	-	-	3	2.8
MP	108	-	-	2	2	1.85
MZ	108	8	-	2	10	9.26
OP	72	1	1	-	2	2.8
PK	72	1	7	-	8	11.1
PL	108	3	3	-	6	5.6
PM	108	17	-	1	18	16.7
SK	108	1	1	1	3	2.8
SL	72	-	-	-	0	0
WM	108	2	1	1	4	3.7
WP	107	4	1	1	6	5.6
ZP	108	9	1	3	13	12
Total	1601	63	23	14	100	6.2

^a^*DS* Dolnośląskie, *KP* Kujawsko-Pomorskie, *LB* Lubelskie, *LD* Łódzkie, *LS* Lubuskie, *MP* Małopolskie, *MZ* Mazowieckie, *OP* Opolskie, *PK* Podkarpackie, *PL* Podlaskie, *PM* Pomorskie, *SK* Świętokrzyskie, *SL* Śląskie, *WM* Warmińsko-Mazurskie, *WP* Wielkopolskie, *ZP* Zachodniopomorskie

### Identification of *C. parvum* subtypes

The subtype was successfully identified by PCR amplification with GP60 primers for 82 out of 100 detected parasite strains but was not for the other 18 *C. parvum* strains. Strains from the IIa subtype family dominated with 80 affiliations and strains from the IId subtype were the other 2 successful identifications. The IIaA17G1R1 (*n* = 26) and IIaA17G2R1 (*n* = 24) strains were detected in the greatest numbers. Three subtypes, IIaA21G1R1 (*n* = 1), IIaA18G3R1 (*n* = 1) and IIaA21R1 (*n* = 1), were only sporadically found. The other detected strains were IIaA15G2R1 (n = 10), IIaA16G1R1b (n = 9), IIaA22G1R1 (*n* = 4), IIaA10G1R1 (*n* = 2), IIaA14G2R1 (*n* = 2) and IIdA24G1 (*n* = 2). None of the tested samples contained more than one *C. parvum* GP60 subtype. The ANOVA showed that only *C. parvum* IIaA17G2R1 and IIaA17G1R1 infections occurred at significantly higher frequency than other parasite subtypes (F-test, *F* = 3.39; *p* = 0.0003). The infections caused by IIaA17G2R1 (total number of infections 24) and IIaA17G1R1 (total number of infections 26) dominated in the studied animal population. In the case of the remaining *C. parvum* subtypes (total number of infections from 1 to 10, LSD_0.05_ = 14.1) there were no significant differences observed in the frequency of their prevalence. Mixed infections caused by two or more parasite subtypes were detected neither at the farm nor at animal level.

**Fig. 2:**
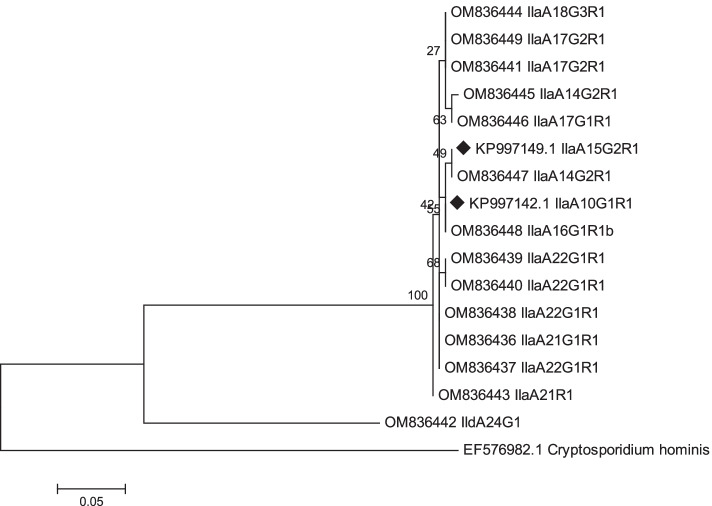
The phylogenetic maximum likelihood tree constructed using the nucleotide sequences (294 bp) of the GP60 gene fragment of *C. parvum* strains detected in cattle. *C. hominis* was used as an outgroup to root the tree. *C. parvum* strains detected in cattle from Poland before 2014 are marked by black diamonds

The nucleotide sequence analysis of the GP60 gene fragment gene showed 100% sequence identity for *C. parvum* strains within the following IIa subtype groups (A10G1R1, A15G2R1, A16G1R1b, A17G1R1, A17G2R1, and A24G1). *C. parvum* IIaA14G2R1 strains revealed 96.4% mutual nucleotide sequence identity. A higher genetic resemblance (99.6–100%) was shown for IIaA22G1R1 strains. The phylogenetic relationships between strains representing different parasite subtypes were presented on Fig. 2.

### Age- and breed-related prevalence of *C. parvum* subtypes in cattle

There was no relationship observed between infections caused by a particular *C. parvum* subtype and cattle age group (Chi-squared test, χ^2^ = 29.4; *p* = 0.133). Nevertheless, particular subtypes were only shown in animals at a certain age. For instance, the IIaA15G2R1, IIaA21G1R1, IIaA10G1R1, IIaA18G3R1, IIaA21R1 and IIaA24G1 subtypes were detected in cattle below 4 weeks of age, while IIaA14G2R1 only occurred in animals between the ages of 4 and 8 weeks (Table 3). Two closely related subtypes, IIaA17G1R1 and IIaA17G2R1, caused infections in calves from all age groups. A significant relationship was shown between the detection of a specific *C. parvum* subtype and cattle breed (Chi-squared test, χ^2^ = 143.6; *p* < 0.001), whereby 10 out of 11 *C*. *parvum* subtypes were found in HO calves, *C. parvum* IIaA22G1R1 strains were only detected in LM cattle and IIaA24G1 only in MM animals (Table 3).

**Table 3:** *C. parvum* subtypes identified in cattle in Poland

*C. parvum* subtype	Number of strains				Total
	by age groups (weeks)			by cattle breeds^a^	
	1–4	> 4–8	> 8–16		
IIaA15G2R1	10	-	-	HO(9), SM(1)	10
IIaA14G2R1	-	2	-	MM(1), HO(1)	2
IIaA16G1R1b	6	-	3	HO(8), MS(1)	9
IIaA17G1R1	10	12	4	HO(16), MDM(7), SM(2), LM(1)	26
IIaA17G2R1	14	6	4	HO(23), MM(1)	24
IIaA21G1R1	1	-	-	HO(1)	1
IIaA22G1R1	3	1	-	LM(4)	4
IIaA10G1R1	2	-	-	HO(2)	2
IIaA18G3R1	1	-	-	HO(1)	1
IIaA21R1	1	-	-	HO(1)	1
IIdA24G1	2	-	-	MM(2)	2
Not identified	11	4	3	MM(2), HO(14), LM(1), SM(1)	18
Total	61	25	14		100

^a^*HO* Polish Black and White Holstein*–*Friesian, *LM* Limousie, *SM* Simentaler breads, *MDM* Mixed dairy-meat breeds, *MM* Mixed meat bread, *MS* Mixed without meat breed

### Geographical distribution of *C. parvum* subtypes

Animals infected with *C. parvum* were detected on 44 (16.48%) of the 267 farms. Two commonly detected IIa subtypes, i.e., IIaA17G1R1 and IIaA17G2R1, were found respectively in 8 and 6 out of the 16 Polish administrative provinces. In comparison to IId subtypes, the two aforementioned strains from the IIa allele families occurred frequently over almost the entire country except in the LS, SL and DS provinces (Fig. 1). They were present on 25 (9.36%) of the investigated farms. The IId subtypes were only found on one farm in the LS province. The relationship between the *C. parvum* subtype and the place of its detection was confirmed by a Pearson’s chi-squared test (χ^2^ = 312.0; *p* < 0.001). The IIaA17G1R1 strains prevailed in PK and SK, while the IIaA17G2R1 did in MP, MZ and WM. On cattle farms in ZP and LS, IIaA16G1R1b and IIdA24G1 infections comprised the majorities of observed infections, respectively.

### Time-related changes in geographical prevalence of *C. parvum* strains

Analysing the structure of the population genetics of *C. parvum* subtypes in cattle in Poland, the disappearance of the strains IIaA14G1R1, IIaA18G1R1c and IIaA19G1R1 was observed with time. In their place, the new variants IIaA18G3R1 and IIaA14G2R1 representing the same genetic family appeared; however, they did so in different geographical locations. Based on the current results, it can be assumed that some subtypes from the IIa (A19G1R1) and IIl (A19R3) genetic families are not currently present in Poland. In the place of the parasite strains IIdA22G1b, IIdA23G1, and IIdA24G1c, which before 2014 had been detected in cattle herds in western Poland (ZP and PM provinces) as well as in LB province in the east of the country, a single subtype IIdA24G1 was found at the time of this investigation. Its appearance was limited to only LS in western Poland. Strains of the IId classification did not spread to other geographical locations in the country, which may indicate their endemic prevalence. For the IIa strains prevalent in Polish cattle, a change in their population dynamics related to the time and place of their detection was observed.

Before 2014, the IIaA15G2R1 subtype was circulating in livestock almost nationwide, also being the only parasite subtype detected in DS, SL, LD, and SK provinces [16]. From 2014 until the conduct of this research, infections caused by *C. parvum* strains of this subtype continued to affect the cattle population in Poland, although their prevalence was limited to only PL and ZP. Also in the same period of time, in the population of IIaA16 strains, two groups of variants were found, i.e., IIaA16G1R1b and IIaA16G3R1. The IIaA16G1R1b subtype prevailed and it was only present in the WP province. Currently, its spread to other regions such as ZP, PK, and PL is observed, which means transmission from the western to the eastern part of Poland. Of note is that infections in cattle caused by this subtype are endemic in the WP province. Over a period of 8 years the frequency of IIaA17G1R1 and IIaA17G2R1 infections was high and they are still considered dominant *C. parvum* subtypes in cattle in Poland. In many cases, they co-circulated in a given geographical area. Furthermore, the constant presence of the IIaA17G1R1 strains in WM, PM and ZP and of the IIaA17G2R1 strains in MZ, PL and WM may indicate the endemic nature of these infections.

## Discussion

Although different *Cryptosporidium* species have been detected in cattle, symptomatic cryptosporidiosis is mainly associated with *C. parvum* infections. The frequency of infections is varied and depends on the animals’ age and the place of breeding [24, 33, 34]. The youngest cattle up to the age of 8 weeks are the most susceptible to infection [4, 16, 35, 36]; therefore, the highest infection rate was observed in animals at that age [19]. The prevalence of *C. parvum* infections in the cattle population in Poland was estimated at 6.2%. As seen in our previous study the overall prevalence of *C. parvum* infections in cattle from Poland at the age of 1 day to 6 years was estimated at 5.1% but only animals below the age of 1 month were the major host for this parasite. In fact, *C. bovis* was the most frequently detected parasite species [15]. Furthermore, similar prevalence rate has been observed in cattle herds in north-eastern France (7.1%), Germany (9.2%) and Sweden (8.1–8.5%) [8, 18, 36, 37] and it was slightly higher than in Romania (5.4%) [38]. Higher prevalences of *C. parvum* in cattle have been reported outside Europe in Argentina (25.5%), Brazil (42.2%) and China (30–60%) [39–41].

It is believed that the animal production system (beef or dairy cattle) could have an impact on the frequency of infection [42, 43]. It is likely that different breeding practices related to animal production systems, for instance a continous calving season for milking cattle rather than periodic calving as in the case of meat breeds, could be considered factors associated with an increase in the number of infections [42]. In this study, *C. parvum* was more often detected in dairy Polish Black and White Holstein Friesian cattle than in meat or mixed dairy–meat animal breeds. This observation is consistent with previous findings that *C. parvum* infections occur more frequently in dairy cattle than in other breeds [42, 44]. Interestingly, it was also among dairy cattle where the most diverse population of *C. parvum* subtypes was found.

Based on the microsatellite sequence analysis of the GP60 gene of *C. parvum* strains isolated from Polish cattle, 11 subtypes representing the IIa and IId genetic families were detected. The six subtypes IIaA10G1R1, IIaA15G2R1, IIaA16G1R1b, IIaA17G1R1, IIaA17G2R1 and IIdA24G1c were previously identified in a bovine host in Poland [16]. Considering the analysed time-related changes on strain prevalence, infections caused by IIaA17G1R1 and IIaA17G2R1 dominate in cattle in Poland and these subtypes often jointly circulate in the same geographical area. In Europe, they have been detected in cattle with frequency varying from 0.4% of IIaA17G1R1 in Northern Ireland [35] to 14.5% in the case of IIaA17G2R1 in Italy [17]. However, they are not considered the dominating *C. parvum* subtypes in European cattle herds [33]. In contrast to the Polish situation, the most widespread subtype in livestock in Europe is IIaA15G2R1 [20, 23, 26, 43, 45]. It is worth noting that the IIaA15G2R1 strains together with IIaA16G1R1b constituted the second largest population of detected strains in cattle in Poland. Strains of the IIaA16G1R1b subtype of *C. parvum* as well as other closely related strains such as IIaA16G3R1 and IIaA16G2R1 have also been identified in livestock in Europe [33, 34]. In the group of IIaA16 variants in Poland, IIaA16G1R1b superabounded. Its presence has been revealed in cattle in Sweden [46] and in Serbia and Montenegro, where its occurrence was considerably heavier than that of other *C. parvum* strains [47]. In Europe, particularly in its south-eastern part, IIaA16G1R1 strains seem to be much more prevalent in cattle [3, 4, 48, 49].

In this study, five new *C. parvum* subtypes (A21R1, A18G3R1, A22G1R1, A21G1R1 and A14G2R1) from the IIa genetic family were detected. They had not been identified in calves in Poland prior to this. The strain IIaA18G3R1 has been previously found in livestock elsewhere in Europe [7, 35]. Its occasional presence has been reported in cattle in the Netherlands (0.8%), while a higher 5.7% prevalence rate has been observed in Spain [7, 22]. This strain was the most abundant in animals in Northern Ireland [35]. Interestingly, its common prevalence has been demonstrated in cattle and humans in Australia [50, 51]. In the case of *C. parvum* IIaA14G2R1, its prevalence in cattle in Europe has not exceeded 3.8% [7, 20, 23, 43]. In the group of newly detected strains in Poland is the one designated IIaA21R1. Surprisingly, elsewhere in Europe it has only been found in humans, these infections having been in Sweden and Norway [52]. The other two new subtypes IIaA22G1R1 and IIaA21G1R1 have been identified in cattle in Germany, Estonia, Sweden and Argentina [18, 20, 53, 54].

There was no relationship observed between infections caused by a specific parasite subtype and cattle age, and this observation is consistant with our previous finding [16]. However, a relationship between a subtype and its geographical prevalence in Poland was demonstrated. The IIaA17G1R1 strains were most frequently noted in cattle in southern Poland, while predominantly IIaA17G2R1 was found on farms located in southern, central and north-eastern Poland. Investigating epidemiology of *Cryptosporidium* infections in other species of farm ruminants in Poland, IIaA17G1R1 strain has also been occasionally detected in sheep [55]. Furthermore, the endemic presence of *C. parvum* IIaA16G1R1, IIaA19G1R1 and IIaA23G1R1 subtypes limited to one farm has been described by Del Coco et al. [53]. The authors did not indicate possible factors which could have contributed to this phenomenon. It is likely that other factors then age or animal breed could have an influence on diversity of detected *C. parvum* subtypes in cattle. Nevertheless, herd size, animal movement, farm management practices, or husbandry system could have an influence on regional subtype prevalence. However, based on the existing data, it would be difficult to determine their significance in the epidemiology of bovine cryptosporidiosis at subtype level.

Population dynamicity in *C. parvum* strains in cattle from Poland has been shown, as exemplified by the emergence of the IIlA19R3 subtype in the bovine host for the first time. In fact, the strains from the genetic family IIl rarely appear in Europe [4, 7, 47]. In the group of *C. parvum* strains which are disappearing from Poland are IIaA14G1R1, IIaA18G1R1c and IIaA19G1R1. Hitherto, the IIaA14G1R1 and IIaA19G1R1 subtypes have been identified in cattle in Estonia [54] and the Netherlands [7, 56] with frequencies of ≤ 7.9% (IIaA14G1R1) and ≤ 4.2% (IIaA19G1R1). In the case of IIaA19G1R1, it has usually been sporadically detected in cattle in Europe with infection prevalence not exeeding 3.9% [7, 23, 27, 54]. Elsewhere than Poland, IIaA18G1R1c strains were only found in diarrhoeic cattle in Sweden [46]. In Poland, *C. parvum* strains from the IId genetic family are the majority of those disappearing or found to occur endemically in particular geographical areas. Out of three IId subtypes circulating before 2014 in cattle, only one remained, IIdA24G1. It had not been identified in cattle in Europe, although it was present in humans [57–59]. However, a similar strain, IIdA24G1c, has been detected in Swedish cattle [46]. Nevertheless, the IIdA16G1b, IIdA17G1d, IIdA19G1, IIdA24G1c and IIdA26G1b bovine *C. parvum* subtypes from the IId genetic family were found in Europe with a frequency ranging from 1.1 to 15.3% [46].

As a final observation, the research findings of this study were limited by sample representativeness and lack of subtype identification for all detected *C. parvum* strains. Although the sampling scheme covered all Polish provinces, the number of sampled animals may not reflect their actual population size perfectly in each sampled region. Therefore, the findings for the variability of detected subtypes in a particular region may be biased and may not mirror the subtypes’ real transmission dynamics in cattle. A further shortcoming is that PCR amplification with GP60 primers was unsuccessful for 18 *C. parvum* strains, probably because of either the low quantity of *Cryptosporidium* DNA or low specificity of the primer’s sequences to the DNA sequence of the detected subtype. A greater number of identified strains could have had influence on the frequency of those strains’ detection and on the interpretation of subtype prevalence results. In particular this could be important for sporadically detected subtypes as it might have changed our perception of their prevalence.

## Conclusions

The results of this study have enhanced our knowledge of the population genetics of *C. parvum* strains circulating over a period of 8 years in Polish cattle herds. Two strain subtypes predominated in cattle, i.e., IIaA17G1R1 and IIaA17G2R1, which over time were not displaced by other subtypes. Nevertheless, a certain strain population dynamicity was observed, which was associated with the disappearance of some strains (IIaA14G1R1, IIaA18G1R1c, IIaA19G1R1 and IIlA19R3) and emergence of new variants (IIaA18G3R1, IIaA14G2R1 and IIdA24G1) from the same genetic family in different geographical locations. The geographical change of strain prevalence from the western to the eastern part of Poland was evident for IIaA16G1R1b subtype. In the case of IIaA15G2R1 its prevalence range was geographically limited although it was circulating in livestock almost nationwide. Strains of the IId subtypes did not spread over time to other geographical locations suggesting their endemic prevalence. No age-dependent distribution of *C. parvum* subtypes was shown among the various cattle breeds being reared in Poland. Breed-dependent diversity of *C. parvum* subtypes was  found, however, with the majority of subtypes being detected in HO cattle. The results of this study will also help in better understanding the subtype-related epidemiology of *C. parvum* infections in cattle and humans that could have originated from livestock, although they do not provide evidence for their possible transmission between hosts.

## Materials and methods

### Cattle faeces

In total, 1601 faecal samples were collected from healthy cattle at the age of 1 week to 4 months from 2014 to 2018 (Table 4). Farms (267) were randomly selected for visits in different locations across each of the 16 administrative provinces of Poland. The sampled animals were divided into three age groups: 1–4 weeks old (*n* = 527), > 4–8 weeks old (*n* = 464), and > 8–16 weeks old (*n* = 610) and were dairy varieties (Polish Black and White Holstein Friesian (HO), Jersey (JE), Polish Red and White Holstein Friesian (RW), Brown Swiss (BS), and Mixed exclusively dairy breed (MS)), meat varieties (Mixed exclusively meat breed (MM), Aberdeen Angus (AN), Charolaise (CH), Salers (SL), Limousie (LM), and Belgian Blue (BB)) and dairy–meat varieties (Mixed dairy–meat breed (MDM), Black and White lowland (NCB), Polish Red (RP), Montbeliarde (MO), Polish Black and White (ZB), and Simentaler (SM)). Freshly voided faecal samples of 10–15 g were placed individually into plastic containers, labelled and sent to the laboratory. For comparison studies analysing the time-related changes of geographical distribution of *C. parvum* subtypes in cattle herds in Poland, archive data on the occurrence in the Polish cattle population of specific subtypes were accessed. Those subtypes were A17G1R1, A17G2R1, A15G2R1, A16G1R1b, A10G1R1, A14G1R1, A16G3R1, A18G1R1c and A19G1R1 from IIa; A23G1, A24G1c and A22G1b from IId; and A19R3 from IIl genetic families. These strains were detected between 2010 and 2014 in different administrative provinces across Poland [16].

**Table 4:** The number of sampled cattle in association to their origin (province)

Province	Number of animals in age groups (weeks)		
	**1–4**	** > 4–8**	** > 8–16**
Lubuskie (LS)	58	22	28
Pomorskie (PM)	78	8	22
Zachodniopomorskie (ZP)	53	29	26
Śląskie (SL)	4	30	38
Opolskie (OP)	8	22	42
Dolnośląskie (DS)	14	32	44
Podkarpackie (PK)	5	31	36
Świętokrzyskie (SK)	43	45	20
Łódzkie (LD)	33	25	50
Małopolskie (MP)	18	26	64
Lubelskie (LB)	46	41	21
Podlaskie (PL)	23	39	46
Mazowieckie (MZ)	46	29	33
Kujawsko-Pomorskie (KP)	27	34	47
Wielkopolskie (WP)	35	21	51
Warmińsko-Mazurskie (WM)	36	30	42
**Total:**	**527**	**464**	**610**

### Detection of *C. parvum* in cattle and subtype identification

Samples were analysed using molecular methods according to previously described protocols [15]. Briefly, parasite genomic DNA was extracted from 0.1 g of faeces using a modified alkali wash and heat lysis method [15, 60]. Identification of *C. parvum* was performed using a PCR with primers targeting the 18 SSU rRNA locus [61] followed by a restriction fragment length polymorphism (RFLP) analysis of the obtained 849 bp amplicons [62, 63]. Extracts of DNA positive for *C. parvum* were subsequently used for subtyping of parasite strains by two nested GP60-PCR assays [64, 65] generating amplicons of approximately 800 bp or 400 bp respectively. Briefly, PCR reactions consisted of the following pre-mixed reagents: 300 µM of each of the four dNTPs, MgCl_2_ at either 1.5 or 3 mM, 1.25 U of KAPA Taq EXtra DNA polymerase in 1 × PCR buffer (Kapa Biosystems), 20 µg BSA (Thermo Scientific^TM^), and molecular grade water up to 50 µl reaction volume. Primers were used at the concentration of 0.1 µM (GP60 assays) or 0.5 µM each (18 SSU rRNA assay). Five or two µl of sample DNA or PCR products were the templates for primary and secondary amplifications respectively. Depending on the assay, the temperature profile was consisting of initial denaturation performed at 94 °C for 3–5 min, then 35 cycles of denaturation at 94 °C for 45 s., annealing at 55 °C (18SSU assay) or 50 °C /52 °C (GP60 assays) for 45 s., and extension at 72 °C for 1 min. A final extension step (72 °C for 7–10 min. depending on the assay) was included followed by a soak at 4 °C. All reactions were performed in a Biometra thermocycler (TProfessional BASIC). For each set of samples, the appropriate positive and negative controls were included during the nucleic acid extraction and PCR analyses. For restriction fragment analysis, 4 μl of nested-PCR product was digested in a total reaction volume of 10 µl consisting of 1 µl *Nde*I or *Mbo*II (Thermo Scientific^TM^) and the appropriate FastDigest reaction buffer at 37 °C for 1 h followed by 65 °C for 5 min. PCR products and their restriction fragments were analysed in 2.5% agarose gels stained with SimplySafe (Eurx).

The GP60 amplicons were excised from the agarose gel, purified and directly sequenced in both directions using the ABI Prism BigDye Terminator v3.1 Cycle sequencing kit on an ABI 3730XL DNA sequencer (Life Technologies, Carlsbad, CA, USA) at the Genomed S.A. sequencing service. Particular subtypes were identified based on trinucleotide repeats present in the analysed sequences. A single representative sequences for each subtype group sharing mutual 100% sequence similarity as well as all sequences that did not show 100% genetic resemblance within particular subtype group were deposited in the GenBank under the accession numbers OM836436—OM836449. The sequence similarity of detected strains was assessed by the maximum likelihood method with a Tamura-Nei parameter model using MEGA version 7.0.9 [66]. The reliability assessment of a topology (bootstrap) of the phylogenetic tree was performed at 1,000 replicates.

### Statistical analyses

The significance of differences in the frequency of *C. parvum* infections between age groups of animals was determined by a chi-square (χ^2^) test. The frequency of parasite occurrence in cattle in different Polish regions (provinces) was analysed using a Kruskal–Wallis Chi-squared test. The relationship between the occurrence of *C. parvum* infection and breed expressed as true prevalence estimate (TPI) was evaluated based on Bayesian posterior probability (POPR) [67]. Estimates for each breed were shown for minimal and maximal POPR thresholds. A difference was regarded as statistically different when POPR was either smaller than 0.05 or grater than 0.95. Data on the sensitivity and specificity of the molecular method employed for parasite detection was included in the model to assess its influence on the estimates [68].

Concluding the statistical work, the χ^2^ test was also employed to show the relationships between *C. parvum* subtypes and their occurrence in calves of different breeds and ages by group and between subtypes and their geographical distribution in Poland. The frequency of occurrence of *C. parvum* subtypes in the studied cattle population was assessed by ANOVA. The calculations were performed with Statgraphics Centurion v. XVI (Statpoint Technologies, Warrenton, VA, USA) and R software v. 4.1.1 [69]. 

## Data Availability

The sequences of GP60 gene of *C. parvum* strains were deposited into NCBI GenBank under accesion numbers OM836436—OM836449.

## Abbreviations

*C. parvum*: *Cryptosporidium parvum*
PCR: Polymerase chain reaction
18SSU rRNA: Small subunit rRNA gene
RFLP: Restriction fragment length polymorphism
GP60: 60-KDa glycoprotein gene
